# Metabolic and nutritional responses of Nile tilapia juveniles to dietary methionine sources

**DOI:** 10.1017/S0007114521001008

**Published:** 2022-01-28

**Authors:** Rita Teodósio, Sofia Engrola, Miguel Cabano, Rita Colen, Karthik Masagounder, Cláudia Aragão

**Affiliations:** 1 Aquaculture Research Group, Centre of Marine Sciences (CCMAR), 8005-139 Faro, Portugal; 2 Universidade do Algarve, 8005-139 Faro, Portugal; 3 Evonik Operations GmbH, 63457 Hanau-Wolfgang, Germany

**Keywords:** Nile tilapia, Methionine sources, Methionine metabolism, Methionine cycle

## Abstract

Commercial diets for tilapia juveniles contain high levels of plant protein sources. Soybean meal has been utilised due to its high protein content; however, soy-based diets are limited in methionine (Met) and require its supplementation to fulfil fish requirements. dl-Methinone (dl-Met) and Ca bis-methionine hydroxyl analogue (MHA-Ca) are synthetic Met sources supplemented in aquafeeds, which may differ in biological efficiency due to structural differences. The present study evaluated the effect of both methionine sources on metabolism and growth of Nile tilapia. A growth trial was performed using three isonitrogenous and isoenergetic diets, containing plant ingredients as protein sources: DLM and MHA diets were supplemented on equimolar levels of Met, while REF diet was not supplemented. Hepatic free Met and one-carbon metabolites were determined in fish fed for 57 d. Metabolism of dl-Met and MHA was analysed by an *in vivo* time-course trial using ^14^C-labelled tracers. Only dl-Met supplementation significantly increased final body weight and improved feed conversion and protein efficiency ratios compared with the REF diet. Our findings indicate that Met in DLM fed fish follows the transsulphuration pathway, while in fish fed MHA and REF diets it is remethylated. The *in vivo* trial revealed that ^14^C-dl-Met is absorbed faster and more retained than ^14^C-MHA, resulting in a greater availability of free Met in the tissues when fish is fed with DLM diet. Our study indicates that dietary dl-Met supplementation improves growth performance and N retention, and that Met absorption and utilisation are influenced by the dietary source in tilapia juveniles.

Tilapia is the second most farmed fish worldwide, just after carp^(1)^. Nevertheless, there are still many challenges to overcome in order to farm tilapia under economic and environmentally sustainable principles. Part of the challenge is dependent on the development of sustainable feeds that will ensure an optimal nutritional status for the fish while promoting N and P retention, thus reducing nutrient excretion into the aquatic environment.

In order to increase the sustainability of aquaculture feeds, inclusion levels of plant ingredients in fish diets have increased. Soybean meal has been successfully included in aquafeeds^(2–4)^ due to its high protein content and relatively well-balanced amino acid profile. However, soy-based diets are limited in methionine (Met) and require its supplementation in order to fulfil fish requirements^(5)^.

Met is an indispensable amino acid involved in protein synthesis, transmethylation reactions and antioxidant defence. Met metabolism occurs mainly in the liver where l-Met is converted into s-adenosylmethionine (SAM), a methyl donor for several reactions. Subsequently, SAM donates its methyl group and is transformed into s-adenosylhomocysteine (SAH), which is rapidly converted into homocysteine. Hepatic homocysteine can be remethylated back to Met by adding a methyl group from trimethylglycine or can irreversibly enter the transsulphuration pathway^(6)^. The transsulphuration pathway converts homocysteine into cystathionine and then cysteine. Ultimately, cysteine can be incorporated into proteins, metabolised into glutathione or oxidised to form taurine^(7)^.

dl-Methionine (dl-Met) and methionine hydroxyl analogue (MHA; dl-2-hydroxy-4-methylthiobutyrate or dl-HMTBA and its Ca salt) are synthetic sources of Met that are often supplemented to animal feeds. dl-Met is a racemic mixture of d- and l-isomers of Met, while MHA is a racemic mixture of d- and l-isomers of MHA^(8)^. MHA chemical structure is similar to that of Met; however, it contains a hydroxyl group instead of the amino group. Recently, studies in rainbow trout (*Oncorhynchus mykiss*) suggested that dl-Met and MHA uptake in the gut apical surface is facilitated by Na-dependent transporters and mediated by proton-independent transporters across the basolateral membrane^(9,10)^. Data comparing the intestinal flux rates of dl-Met and MHA suggest a faster intestinal transport of the former synthetic source^(8,10,11)^.

Since animals can only metabolise l-amino acids, d-isomers of dl-Met first need to be converted to a keto-Met intermediate (keto-methylthio-butanoic acid, KMB) and then transaminated to l-Met before becoming available^(8,12)^. On the other hand, both the d- and l-isomers of MHA need to be converted to KMB to become available^(8,12)^. These differences are likely to be reflected as differences in absorption and metabolism and may result in different biological efficiencies. Numerous studies in terrestrial animals and fish advocate that MHA supplementation results in lower bioavailability of this compound compared with dl-Met^(13–15)^. In rainbow trout, dietary MHA-Ca salt was found to have lower bioavailability than dl-Met, resulting in 69 % lower fish weight gain, 60 % lower growth rate and 73 % lower N retention in fish^(16)^. Similarly, growth performance and feed utilisation indicators in common carp (*Cyprinus carpio*) fed dl-Met and MHA-Ca supplemented diets demonstrated that MHA-Ca was 41–50 % as available as dl-Met on weight-for-weight basis^(17)^. In contrast, some authors report similar efficiencies among Met sources based on growth and feed efficiency in several aquatic animals^(18–21)^. On the other hand, a study performed in channel catfish observed that dietary MHA-Ca supplementation resulted in improved body weight, weight gain, feed conversion ratio and protein efficiency ratio^(22)^. Based on the data published, NRC^(23)^ concluded that it is reasonable to assume that the biological efficacy of MHA in fish is 75–80 % that of dl-Met on an equimolar basis (63–67 % on a weight basis). Generating data on growth, diet utilisation and Met metabolism in a commercial relevant species such as the Nile tilapia (*Oreochromis niloticus*) is of paramount importance for the aquaculture industry.

In this context, the objective of the present study was to understand how the dietary source of Met affects Met metabolism and growth of Nile tilapia juveniles. Radiolabelled Met sources were used in a nutrient flux assay to evaluate their influence in the amino acid metabolic pathways.

## Materials and methods

### Diets

Three experimental diets were formulated to be isonitrogenous and isoenergetic (Table 1). Diet REF was a negative control, without fishmeal inclusion, formulated to be 40 % below the Met requirement for Nile tilapia^(23)^. No Met was supplemented to this diet. Experimental diets were the REF diet supplemented with 1·5 g/kg dl-Met (dlM diet) and 1·8 g/kg MHA-Ca (equal on molar basis to 1·5 g/kg dl-Met; MHA diet). To minimise variability among diets, one common basal diet was formulated, and the respective supplemental Met source was added in DLM and MHA diets. Diets were formulated to meet the minimum requirements of amino acids on digestible basis for Nile tilapia juveniles, except for Met. Apparent digestibility coefficients of amino acids for the ingredients used were taken from published review data^(24,25)^. Diets were supplemented with selected indispensable amino acids and di-calcium phosphate to avoid amino acid or mineral imbalances.

**Table 1.** Formulation and proximate composition of the experimental diets (g/kg diet)

	Dietary treatments		
Ingredients	REF	DLM	MHA
Soybean meal *	350·0	349·5	349·4
Soy protein concentrate †	92·5	92·4	92·3
Maize meal ‡	299·2	298·8	298·7
Pea protein concentrate §	77·8	77·7	77·7
Wheat bran ||	66·3	66·2	66·2
Soybean oil ¶	48·0	47·9	47·9
Fish oil **	20·0	20·0	20·0
Di-calcium phosphate ††	30·0	30·0	29·9
Vit–min premix ‡‡	10·0	10·0	10·0
l-Lysine sulphate	3·1	3·1	3·1
l-Threonine	1·9	1·9	1·9
l-Tryptophan	0·6	0·6	0·6
l-Histidine	0·6	0·6	0·6
dl-Methionine	0·0	1·5	0·0
Ca bis-methionine hydroxyl analogue	0·0	0·0	1·8
Analysed proximate composition (as fed basis)			
DM	941·6	919·7	950·5
Ash	58·3	61·2	62·8
Crude protein	326·4	323·1	323·9
Crude fat	88·2	89·3	95·3
Total P	7·3	7·9	8·1
Gross energy (MJ/kg)	19·0	18·6	19·2

* Solvent extracted dehulled soybean meal: 457 g/kg crude protein (CP), 31 g/kg crude fat (CF), CARGILL.

† Soycomil P: 620 g/kg CP, 7 g/kg CF, ADM.

‡ Maize meal: 81 g/kg CP; 37 g/kg CF, Casa Lanchinha.

§ Lysamine GPS: 840 g/kg CP, 10 g/kg CF, ROQUETTE Frères.

|| Wheat bran: 149 g/kg CP, 40 g/kg CF, Cerealis Moagens S.A.

¶ Henry Lamotte Oils GmbH.

** Sopropêche.

†† DCP: 168 g/kg P, 209 g/kg Ca, Premix Lda.

‡‡ PREMIX Lda. Vitamins (mg/kg diet): dl-alpha tocopherol acetate, 100 mg; sodium menadione bisulphate, 25 mg; retinyl acetate, 6.88 mg; dl-cholecalciferol, 0.050 mg; thiamin, 30 mg; riboflavin, 30 mg; pyridoxine, 20 mg; cyanocobalamin, 0.1 mg; nicotinic acid, 200 mg; folic acid, 15 mg; ascorbic acid, 1000 mg; inositol, 500 mg; biotin, 3 mg; calcium panthotenate, 100 mg; choline chloride, 1000 mg; betaine, 500 mg. Minerals (g or mg kg/diet): cobalt carbonate, 0·65 mg; copper sulphate, 9 mg; ferric sulphate, 6 mg; potassium iodide, 0·5 mg; manganese oxide, 9·6 mg; sodium selenite, 0·01 mg; zinc sulphate, 7·5 mg; sodium chloride, 400 mg; calcium carbonate, 1·86 g; excipient wheat middlings.

Upon ingredient grinding with a hammer mill (model SH1; Hosokawa-Alpine) and its mixing in a double-helixmixer, all diets (pellet sizes 1·2 and 2·0 mm) were manufactured using a twin-screw extruder (model BC45; Clextral) at SPAROS Lda. Upon cold extrusion, diets were dried in a vibrating fluid bed dryer (model DR100; TGC Extrusion). After cooling, the oils were added to the pellets by vacuum coating (model PG-10VCLAB). Throughout the duration of the trial, experimental feeds were stored at room temperature, in a cool and aerated storage room. Proximate composition and amino acid analysis were determined in all experimental diets, as reported in Tables 1 and 2, respectively.

**Table 2.** Amino acid and Ca bis-methionine hydroxyl analogue (MHA-Ca) content of experimental diets (g/kg diet)

Analysed values	Dietary treatments		
	REF	DLM	MHA
Lysine	17·8	19·7	19·1
Methionine	4·4	5·6*	4·4
Cysteine	4·4	4·3	4·4
Threonine	13·5	13·6	13·8
Arginine	23·0	23·3	23·9
Isoleucine	13·9	14·0	14·3
Leucine	25·2	25·2	25·7
Valine	15·2	15·2	15·7
Histidine	8·3	8·5	8·7
Phenylalanine	15·9	16·0	16·3
Glycine	13·3	13·3	13·7
Serine	16·0	15·7	16·2
Proline	17·1	16·7	17·1
Alanine	14·4	14·3	14·7
Aspartate	34·2	34·4	35·4
Glutamate	56·7	55·9	57·3
MHA-Ca†	0·0	0·0	1·3
Taurine	<0·1	<0·1	<0·1

* Analysed dl-Met in the DLM diet was 1.1 g/kg.

† Active content of MHA in the MHA diet is 1.1 g/kg (equal on molar basis to dl-Met) since MHA-Ca active form is 84 %.

### Growth trial

The experiment was carried out in compliance with the Guidelines of the European Union Council (Directive 2010/63/EU) and Portuguese legislation for the use of laboratory animals. Nile tilapia (Silver Natural Male Tilapia™) juveniles were obtained from Til-Aqua International B.V. and the experiment was conducted at Centre of Marine Sciences of Algarve (CCMAR) facilities (Faro, Portugal). CCMAR facilities and their staff are certified to house and conduct experiments with live animals (Group-C licenses by the Direção Geral de Alimentação e Veterinária, Ministério da Agricultura, Florestas e Desenvolvimento Rural, Portugal). Upon arrival, fish were acclimatised to the new rearing facilities in a recirculating aquaculture system and were fed a commercial diet (crude protein: 340 g/kg; crude fat: 50 g/kg).

Juvenile Nile tilapia were reared in 100-litre cylindrical tanks in a recirculating aquaculture system equipped with a mechanical filter, a submerged biological filter and a UV steriliser. Photoperiod was natural (10 h light: 14 h dark), temperature averaged 25·9 (sd 0·5)°C and dissolved oxygen in water was maintained above 85 % of saturation. Water quality parameters were monitored daily and adjusted when necessary: pH was maintained between 7·0 and 8·2 and the concentration of unionised ammonia and nitrites in water was 0 mg/l during the whole experimental period. Mortality was monitored daily.

Fish with an initial mean body weight of 2·3 (sd 0·4) g were allocated into nine tanks at an initial density of 1·1 kg/m^3^ (fifty fish per tank). Eight fish from the initial stock were sampled and the whole fish were stored at −20°C until analysis of proximate composition. Triplicate tanks were randomly assigned to one of the three dietary treatments (REF, DLM and MHA). Fish were fed to visual satiety by hand, three times a day (09.30, 12.30 and 16.30 hours) and feed intake was recorded daily for 57 d.

At the end of the trial, each tank was bulk weighed. Ten fish from each tank were euthanised with a lethal dose of anaesthetic (1·5 ml/l phenoxyethanol; Sigma-Aldrich). Whole-body, liver and viscera weight of five individual fish were recorded for calculation of biometric indexes and liver samples were snap-frozen in liquid N_2_ and kept at −20°C until free amino acid analysis. The other five fish were stored at −20°C until analysis of whole-body proximate composition and amino acid content. Fish were fasted for 24 h before initial and final samplings.

### Metabolic utilisation of supplemental methionine sources

At the end of the growth trial, to understand how different dietary Met sources are absorbed and metabolised in tilapia juveniles, a time-course metabolic trial was performed using radiolabelled dl-Met and MHA. REF diet was not included in this trial since the aim was to assess putative differences in the metabolic flux of the Met supplemental sources and not of the intact protein. Random fish fed the DLM or MHA diet were transferred to the nutrient flux laboratory after being fasted for 24 h.

dl-(1–^14^C)-Methionine (^14^C-dl-Met; 0·00185 GBq) and (1–^14^C)-calcium bis-methionine hydroxyl analogue (^14^C-MHA; 0·00185 GBq) (Campro Scientific GmbH) were used as tracers to radiolabel the experimental diets (DLM and MHA). The methodology for labelling the experimental diets was established in previous works of the authors^(26–28)^. The tracers were diluted in freshwater Ringer solution and a known value of the tracer was dispensed using a micropipette on individual pellets of the correspondent experimental diet. The pellets were dried at 50°C for 1 h. Eight pellets per fish, corresponding to 0·3 % body weight, were loaded into a hollow plastic tube of 1·5 mm inner diameter and stored for subsequent tube-feeding. Prior to tube-feeding, the amount of radioactivity (DPM) of ten individual pellets from each experimental diet labelled with the corresponding tracer was determined in a TriCarb 2910TR low activity liquid scintillation analyser (Perkin Elmer) after adding the scintillation cocktail (Ultima Gold XR; Perkin Elmer).

The *in vivo* method of tube-feeding used to perform the metabolic trials was adapted from Costas^(29)^, which was a modification of the method first described by Rust^(30)^ and modified by Rønnestad^(31)^. Fish were anaesthetised (200 mg/l MS-222 buffered with sodium bicarbonate; Sigma-Aldrich) and subsequently were taken out of the water using a fish net and placed onto a dry plastic tray. The previously loaded plastic tube containing the radiolabelled pellets was inserted into the fish mouth and the feed pellets were gently pushed directly into the oesophagus using a solid piece with a smaller diameter placed inside as a plunger. The diameter and length of the hollow plastic tube were previously tested to avoid injuring the fish oesophagus. This procedure lasted approximately 10 s. The metabolic fate of the tracer (^14^C-dl-Met or ^14^C-MHA) was considered to represent the fate of the tracee (dl-Met or MHA-Ca)^(32)^.

After tube-feeding, fish were placed into a tank with clean and aerated freshwater to eliminate any residual anaesthetic and were monitored for eventual pellet regurgitation. After this period, fish were transferred into individual incubation chambers containing 2 litres of freshwater at 26°C. Each chamber was hermetically sealed and supplied with a gentle oxygen flow. After the incubation period, oxygen flow was stopped and fish were euthanised inside the chambers by a lethal dose of anaesthetic (750 mg/l of MS-222 buffered with sodium bicarbonate). The incubation periods were 1, 2, 3, 4 or 6 h (*n* 6–7 fish for each diet and incubation period). Fish was removed from the chamber and weighed.

Water samples were collected from each individual chamber to determine the amount of radioactivity (DPM) present in the incubation water. The radioactivity present in the incubation water resulted from evacuated (non-absorbed) and/or catabolised radiolabelled Met source as CO_2_. Viscera, liver, skin-on fillets and the rest of the fish were collected and weighed. Viscera consisted of washed digestive tract (so that no alimentary bolus was present), spleen, pancreas and perivisceral fat and will be designated from hereafter as *Viscera* compartment; skin-on fillets (muscle with skin), as *Muscle* compartment and the rest of the fish (consisting of head, heart, kidney, bones and fins) as *Residual* compartment. *Viscera* and *Liver* compartments were analysed as whole. *Muscle* and *Residual* compartments were minced using a coffee grinder until a homogeneous mixture was obtained and 0·5 g samples were taken for further analysis.

All fish tissues were incubated at 4°C for 24 h with 6 % (w/v) TCA, with periodical stirrings. After this period, tissue samples were taken and the TCA samples collected for radioactivity determination (from hereafter designated as *Free* fraction). In order to get a better insight of the metabolic flux of ^14^C-dl-Met and ^14^C-MHA, tissue samples from the 6 h incubation period were homogenised and underwent a series of extraction procedures to separate organic compounds such as protein, lipids and other metabolites as described previously by Rocha^(26)^. Briefly, samples were transferred to a clean vial and further homogenised in distilled water using an Ultra-turrax homogeniser (IKA). Total lipids were extracted using a modified Bligh and Dyer method^(33)^ for small volumes, and total protein was extracted based on a TCA precipitation method^(34)^. The supernatant containing non-extracted metabolites was collected for radioactivity determination. Protein pellet was resuspended in an appropriate volume of Solvable™ (Perkin Elmer) and kept at 50ºC until complete solubilisation was achieved. Lipids and other metabolites are designated as *Others* fraction, while protein as *Protein* fraction. Scintillation cocktail (Ultima Gold XR; Perkin Elmer) was added to all samples and DPM were counted in a TriCarb 2910TR low activity liquid scintillation analyser (Perkin Elmer). All counts were corrected for quench and lumex. Radioactivity found in the *Incubation Water* and in the *Free* fractions of all compartments was normalised for fish or tissue weight and expressed as DPM/g. Radioactivity present 6 h after feeding the radiolabelled nutrients in the *Protein* and *Others* fractions of all compartments was expressed as DPM.

To estimate the availability of the radiolabelled nutrient in each compartment, the area under the curve (AUC) from 1 to 6 h was calculated as follows:

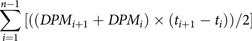




where *t* is the time point, *DPM*_*i*_ is the amount of radioactivity found at *t*_*i*_ and *n* is the total number of measures^(35)^. AUC was expressed as a percentage of total cumulated (1–6 h) radioactivity (DPM) per dietary treatment.

### Biochemical analysis

Raw materials (soybean meal, soy protein concentrate, pea protein concentrate, maize meal and wheat bran) were analysed for DM, crude protein and amino acid content using NIR (AMINONIR^®^, Evonik Nutrition & Care) before diet formulation.

Chemical analysis followed standard procedures of the Association of Official Analytical Chemists^(36)^ and was run in duplicates. Before analysis, diets and pooled whole-body fish were finely ground. Moisture content was determined by drying the samples at 105°C for 24 h and ash content by incineration in a muffle furnace at 550°C for 6 h. Freeze-dried whole-body samples and diets were analysed for crude protein (N × 6·25) by wet chemistry (AMINOLab^®^, Evonik Nutrition & Care) using the combustion/Dumas method; crude fat by petroleum ether extraction using a Soxtherm Multistat/SX PC (Gerhardt); gross energy by combustion in an adiabatic bomb calorimeter (Werke C2000; IKA) calibrated with benzoic acid and P content by digestion at 230°C in a Kjeldatherm block digestion unit followed by digestion at 75°C in a water bath and absorbance determination at 820 nm (adapted from AFNOR V 04-406).

Amino acid content in diets and whole-body fish samples were analysed by wet chemistry (AMINOLab^®^, Evonik Nutrition & Care) using ion exchange chromatography. Hepatic free amino acids and SAM and SAH contents were determined after homogenisation of freeze-dried samples in 0·1 M HCl on ice, centrifugation at 1500 × ***g*** at 4°C for 15 min and deproteinisation of the supernatant by centrifugal ultrafiltration (10 kDa cut-off, 2500 × ***g*** at 4°C for 20 min). All samples were pre-column derivatised with Waters AccQ Fluor Reagent (6-aminoquinolyl-N-hydroxysuccinimidyl carbamate) using the AccQ Tag method (Waters), except samples for SAM and SAH analysis, which were not derivatised. All analyses were performed by ultra-high-performance liquid chromatography on a Waters Reversed-Phase Amino Acid Analysis System, using norvaline as an internal standard. Amino acids were identified by retention times of standard mixtures (Waters) and pure standards (Sigma-Aldrich). Instrument control, data acquisition and processing were achieved by the use of Waters Empower software.

### Nutritional indicators

Growth performance parameters, somatic indexes and nutrient retention were calculated as follows:

Daily voluntary feed intake (% ABM/d) = 100 × (apparent feed intake/ABM/d), where ABM is average body mass = (final biomass + initial biomass)/2.

Feed conversion ratio = apparent feed intake/wet weight gain.

Protein efficiency ratio = wet weight gain/crude protein intake.

Hepatosomatic index (%) = 100 × (liver weight/total weight).

Viscerosomatic index (%) = 100 × (viscera weight/total weight).

Protein or energy retention (% intake) = 100 × ((final body protein or energy content – initial body protein or energy content)/(protein or energy intake)).

Daily N intake (mg N/kg/d) = N intake/ABM/d.

Daily N gain (mg N/kg/d) = (final body N content − initial body N content)/ABM/d.

Daily N loss (mg N/kg/d) = daily N intake − daily N gain.

### Statistical analysis

Sample size was determined based on preliminary power analysis to ensure a probability of at least 80 % in the detection of treatment effects. Data are presented as means and standard deviations. Data expressed as a percentage were arcsine square root transformed previously to the statistical analysis^(37)^. All data were checked for normal distribution and homogeneity of variances. Differences among groups were identified by one-way ANOVA followed by Tukey’s multiple-comparison test or, when the assumptions for the ANOVA failed, by Kruskal–Wallis one-way ANOVA by ranks followed by Dunn’s multiple-comparison tests. Data from the time-course metabolic trials were subjected to linear regression analysis to understand the relationship between the tracer (^14^C-dl-Met or ^14^C-MHA) and incubation time in each compartment (*Incubation Water*, *Viscera*, *Liver*, *Muscle* and *Residual* compartments). Additionally, one-way ANOVA followed by planned contrasts test was performed to compare the differences between ^14^C-dl-Met and ^14^C-MHA fed fish at each time point. At 6 h time point, Mann–Whitney *U* test was additionally used to identify differences in the *Protein* and *Others* fractions of the several body compartments. All statistical differences were considered significant at *P* < 0·05. Statistical analyses were performed using the open source software R version 3.6.1.

## Results

### Growth performance and feed utilisation

At the end of the growth trial, survival was 100 % in all treatments and fish had a 9–10-fold increase in body weight (Table 3). Fish fed the DLM diet were significantly heavier (28·3 (sd 4·7) g) than fish fed the REF (24·1 (sd 3·0) g) and the MHA (24·8 (sd 2·7) g) diets (*P* < 0·05). Fish biomass gain differed marginally with the different dietary treatments (*P* = 0·08). Feed intake was similar among experimental groups. Fish fed the DLM diet presented a significantly lower feed conversion ratio than fish fed the REF diet (*P* < 0·05). Protein efficiency ratio was significantly higher in fish fed the DLM (3·4 (sd 0·1)) than the REF (3·1 (sd 0·1)) diet (*P* < 0·05). Dietary treatments did not affect significantly hepatosomatic and viscerosomatic indexes (*P* > 0·05).

**Table 3.** Growth performance, somatic indexes and feed utilisation of Nile tilapia juveniles fed the experimental diets for 57 d*(Mean values and standard deviations)

	Dietary treatments					
	REF		DLM		MHA	
	Mean	sd	Mean	sd	Mean	sd
Final body weight (g)	24·1^b^	3·0	28·3^a^	4·7	24·8^b^	2·7
Biomass gain (g/tank)	928	85	1142	55	1052	118
Daily voluntary feed intake (% average biomass/d)	2·8	0·1	2·7	0·1	2·7	0·1
Feed conversion ratio	1·0^a^	0·0	0·9^b^	0·0	0·9^ab^	0·0
Protein efficiency ratio	3·1^b^	0·1	3·4^a^	0·1	3·3^ab^	0·1
Hepatosomatic index (HSI, %)	1·53	0·47	1·34	0·33	1·30	0·20
Viscerosomatic index (VSI, %)	9·47	0·64	9·07	1·02	8·74	1·37

* Initial body weight = 2·3 (sd 0·4) g for all dietary treatments (*n* 150). Values are presented as means and standard deviations (*n* 15 for final body weight, HSI and VSI; *n* 3 for the remaining parameters).

^a,b^Different superscripts within the same row indicate significant differences (*P* < 0·05) among diets. Absence of superscripts indicates no significant differences.

### Whole-body fish composition and nutrient retention

Fish from the DLM diet presented significantly higher body protein and energy content than fish from the MHA diet (*P* < 0·05; Table 4). Fish whole-body moisture, ash, fat and P content were not affected by the dietary treatments (*P* > 0·05). Additionally, whole-body total Met content was significantly higher in DLM fed fish (3·86 (sd 0·01) g/kg) than in fish from the REF (3·54 (sd 0·11) g/kg) or MHA (3·50 (sd 0·14) g/kg) dietary treatments (*P* < 0·05). No significant differences were found for the other amino acids among experimental groups. Protein and energy retention were significantly higher for fish fed the DLM diet than for fish fed the other diets (*P* < 0·05; Table 4).

**Table 4.** Whole-body composition and protein and energy retention of Nile tilapia juveniles fed the experimental diets for 57 d*(Mean values and standard deviations)

Body composition (g/kg wet weight)	Dietary treatments					
	REF		DLM		MHA	
	Mean	sd	Mean	sd	Mean	sd
Moisture	717·2	2·8	709·8	3·1	725·8	12·6
Ash	36·3	2·3	38·3	0·5	35·8	0·5
Protein	147·8^ab^	3·3	155·9^a^	0·7	145·1^b^	5·4
Fat	82·2	3·3	79·4	1·9	74·2	4·1
P	4·7	0·2	5·1	0·1	4·9	0·2
Energy (MJ/kg)	6·6^ab^	0·1	6·8^a^	0·1	6·3^b^	0·3
Retention (% intake)						
Protein	44·7^b^	2·2	52·2^a^	1·7	47·8^b^	0·8
Energy	35·7^b^	1·2	40·6^a^	1·4	36·1^b^	0·6

* Initial body composition: moisture = 742.8 g/kg WW; ash = 40.0 g/kg WW; protein = 164.6 g/kg WW; fat = 47.7 g/kg WW; P = 5.9 g/kg WW; energy = 5.5 MJ/kg WW. Values are presented as means and standard deviations (*n* 3).

^a,b^Different superscripts within the same row indicate significant differences (*P* < 0.05) among diets. Absence of superscripts indicates no significant differences.

**Fig. 1.**
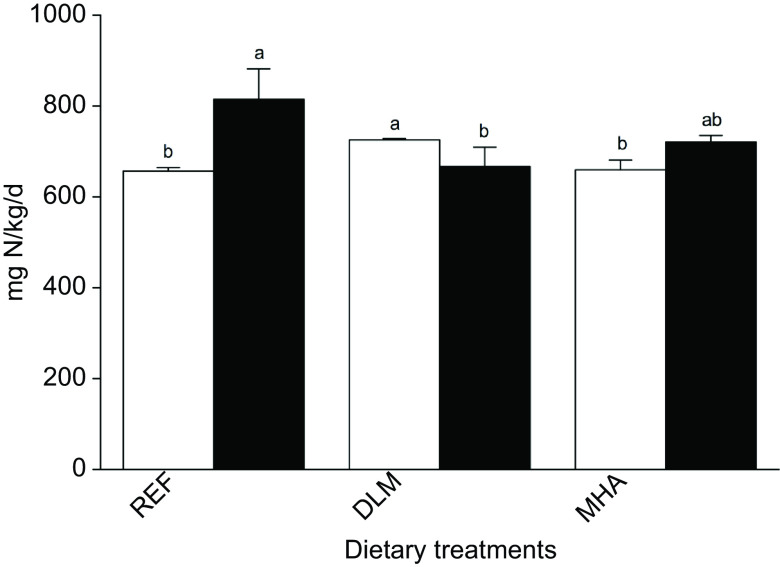
Daily N balance in Nile tilapia juveniles fed the experimental diets for 57 d. Values are presented as means and standard deviations (*n* 3). Different letters within the same compartment indicate significant differences (*P* < 0·05) among diets. 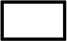, N gain; 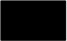, N loss.

Dietary treatments did not influence daily N intake. DLM fish exhibited the highest daily N gain (726 (sd 3) mg N/kg/d), significantly different from REF (657 (sd 8) mg N/kg/d) or MHA (660(sd 22) mg N/kg/d) fed fish (*P* < 0·05). Moreover, fish from the DLM group presented the lowest daily N loss (667 (sd 43) mg N/kg/d), which was significantly different from the REF group (*P* < 0·05; Fig. 1).

### Methionine and one-carbon metabolites

**Fig. 2.**
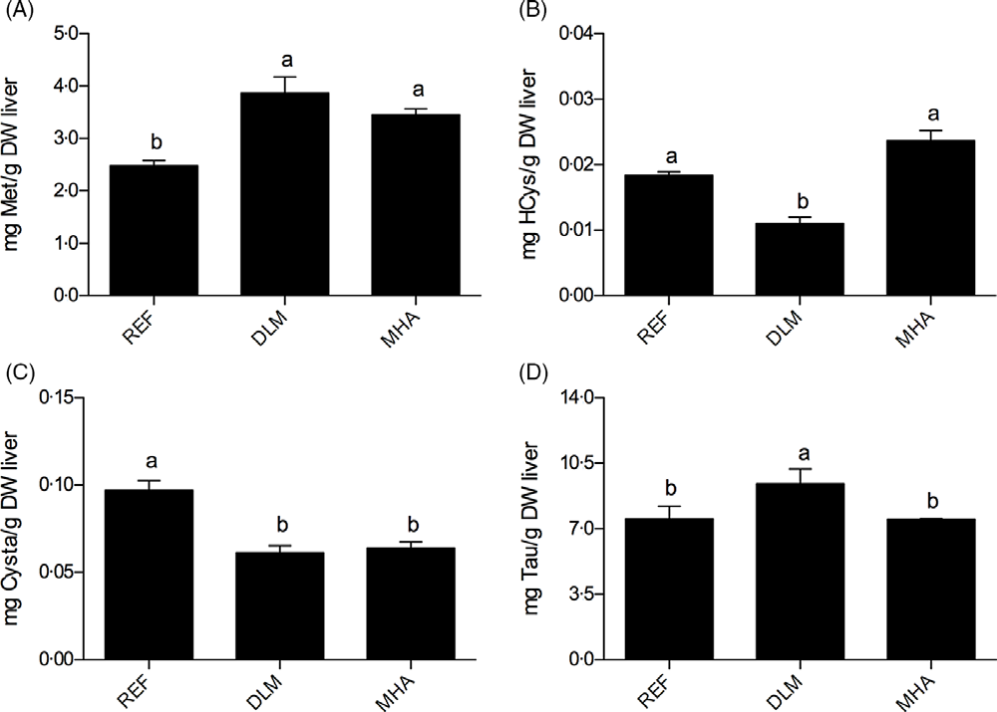
Free methionine (A), homocysteine (B), cystathionine (C) and taurine (D) content in liver of Nile tilapia juveniles fed the experimental diets for 57 d. Values are presented as means and standard deviation (*n* 3). Different letters indicate significant differences (*P* < 0·05) among diets.

Free Met (Fig. 2(A)) was significantly higher in liver of fish fed the DLM and MHA diets than in REF fed fish (*P* < 0·05). SAM (1·25–1·35 mg SAM/g dry weight (DW) liver), SAH (0·23–0·27 mg SAH/g DW liver) or SAM/SAH ratio (4·79–5·48) were not affected by the dietary treatments (*P* > 0·05). Homocysteine levels (Fig. 2(B)) were significantly lower in DLM fish than in fish fed REF and MHA diets (*P* < 0·05). Cystathionine was significantly lower in fish fed the supplemented diets than in fish fed the REF diet (*P* < 0·05; Fig. 2(C)). Free cysteine content was not affected by the dietary treatments (*P* > 0·05; 0·17–0·20 mg Cys/g DW liver). Fish fed the DLM diet had more hepatic taurine content than fish fed REF and MHA diets (*P* < 0·05). In addition, there were no significant differences in taurine content between fish fed REF and MHA diets (*P* > 0·05; Fig. 2(D)). Hepatic trimethylglycine was significantly lower in fish fed the DLM (0·05 (sd 0·00) mg TMG/g DW liver) than the REF (0·06 (sd 0·00) mg TMG/g DW liver) diet (*P* < 0·05). No significant differences in hepatic trimethylglycine content were found for fish fed the MHA diet compared with the other groups (*P* > 0·05).

**Fig. 3.**
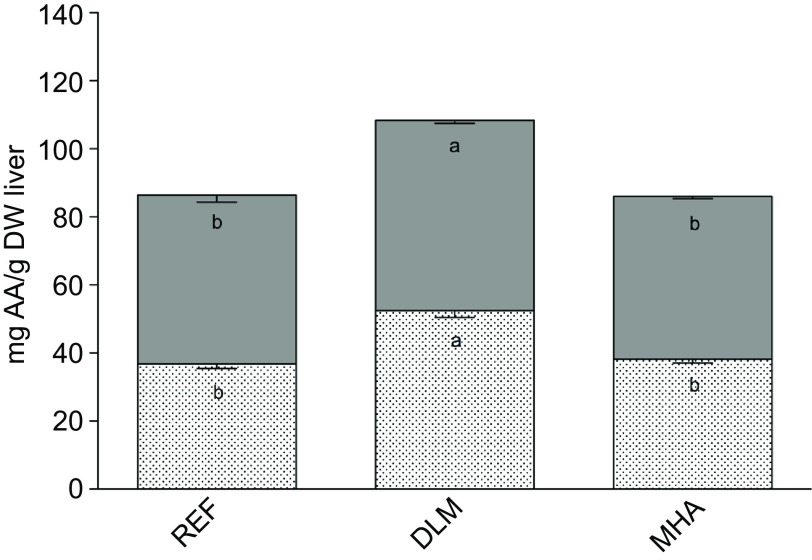
Sum of free indispensable (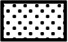, sum IAA) and dispensable (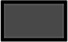, sum DAA) amino acids in liver of Nile tilapia juveniles fed the experimental diets for 57 d. Values are presented as means and standard deviation (*n* 3). Different letters within the same compartment indicate significant differences (*P* < 0·05) among diets.

Moreover, total free amino acids were significantly higher in DLM fish liver (108·42 (sd 2·90) mg/g DW liver) than in liver of fish fed the REF (86·48 (sd 3·29) mg/g DW liver) and the MHA (86·08 (sd 1·81) mg/g DW liver) diets (*P* < 0·05). Similarly, the sum of free indispensable and dispensable amino acids was significantly higher in liver of fish fed the DLM diet than in liver of fish fed the other diets (*P* < 0·05; Fig. 3).

### Metabolic utilisation of supplemental methionine sources

**Fig. 4.**
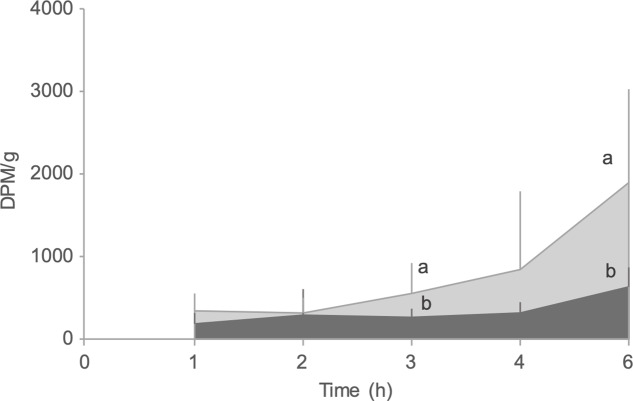
Radioactivity (DPM/g of fish) in the *Incubation Water* compartment at 1, 2, 3, 4 and 6 h after tube-feeding experimental diets labelled with ^14^C-dl-Met or ^14^C-MHA. Values are presented as means and standard deviation (*n* 6–7 fish for each diet and incubation period). Different letters at the same time point indicate significant differences (*P* < 0·05) between diets. 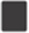, DLM; 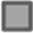, MHA.

The amount of radiolabelled tracer found in *Incubation Water* had a linear increase during the time course for both DLM and MHA treatments (Table 5; *P* < 0·05). Also, at the end of the time course (6 h) a higher amount of ^14^C-MHA was present in the *Incubation Water* compartment than of ^14^C-dl-Met (*P* < 0·05; Fig. 4). Furthermore, the AUC for the MHA diet was 2·4-fold higher than for the DLM diet, indicating a higher evacuation and/or catabolism of ^14^C-MHA compared with ^14^C-dl-Met by juvenile tilapia (Table 5).

**Table 5.** Linear regression analysis and AUC (1–6 h) of the free fractions analysed in the time-course metabolic trial

Compartments	DLM	MHA
Linear regression *P* value		
Incubation Water	<0·001	<0·001
Viscera	0·015	0·489
Liver	0·010	0·222
Residual	0·219	0·002
Muscle	0·821	0·005

AUC* (% total cumulated radioactivity DPM)		
Incubation Water	2·0	4·7
Viscera	54·5	60·0
Liver	29·7	26·1
Residual	8·3	6·3
Muscle	5·5	2·9

* Please refer to ‘Metabolic utilisation of supplemental methionine sources’ section for further details.

**Fig. 5.**
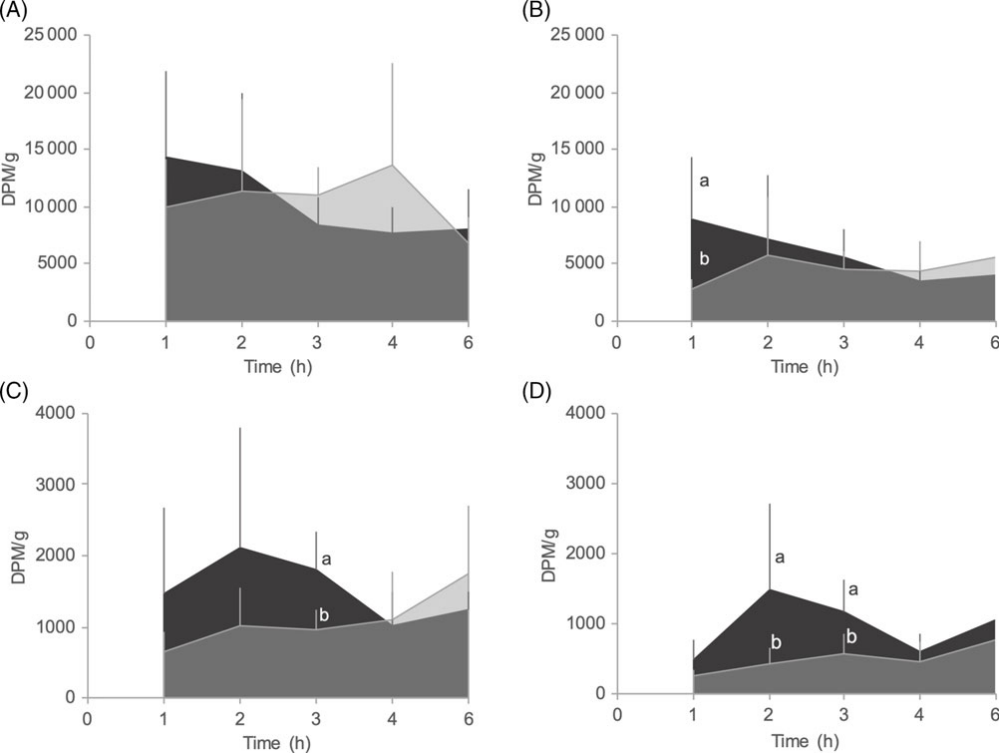
Radioactivity (DPM/g of tissue) in the *Viscera* (a), *Liver* (b), *Residual* (c) and *Muscle* (d) *Free* fractions at 1, 2, 3, 4 and 6 h after tube-feeding experimental diets labelled with ^14^C-dl-Met or ^14^C-MHA. Values are presented as means and standard deviation (*n* 6–7 fish for each diet and incubation period). Different letters at the same time-point indicate significant differences (*P* < 0·05) between diets. 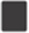, DLM; 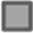, MHA.

After 1 h of diet ingestion, the amount of tracer found in *Viscera* (Fig. 5(A)) and *Liver* (Fig. 5(B)) *Free* fractions was higher in the DLM than in the MHA fish, although only significantly different for the *Liver Free* fraction (*P* < 0·05; Fig. 5(B)). The ^14^C-dl-Met presented a linear decrease during the incubation period in both compartments (*P* < 0·05; Table 5). On the other hand, this linear pattern was not found in fish fed MHA diet. The amount of tracer in *Viscera Free* fraction increased up to 4 h and then decreased at 6 h to values slightly below the values determined for the DLM fed fish (Fig. 5(A)). After 4 h of ingestion, the amount of tracer found in *Viscera Free* fraction of MHA fed fish was significantly higher than in fish fed the DLM diet (*P* < 0·05; Fig. 5(A)). Regarding the ^14^C-MHA in the *Liver Free* fraction, a plateau was observed from 2 h until the end of the incubation period (Fig. 5(B)). The AUC in the *Viscera* and *Liver Free* fractions was similar for both dietary treatments, indicating a similar bioavailability of both Met sources along the experimental period (Table 5).

The presence of ^14^C-dl-Met in the *Residual Free* fraction presented a peak at 2 h after diet ingestion (Fig. 5(C)), while for ^14^C-MHA a linear increase was observed during the whole incubation period (*P* < 0·05; Table 5). The amount of tracer found in the *Residual Free* fraction of DLM fed fish was significantly higher than in fish fed the MHA diet (*P* < 0·05; Fig. 5(C)) at 3 h after diet ingestion. Similar to the *Residual Free* fraction, the amount of tracer found in the *Muscle Free* fraction (Fig. 5(D)) of MHA fed fish exhibited a linear increase with time (*P* < 0·05; Table 5), while for the ^14^C-dl-Met a peak was found 2 h after diet ingestion. The amount of ^14^C-dl-Met found in *Muscle Free* fraction was significantly higher than that of ^14^C-MHA (*P* < 0·05) at 2 and 3 h after diet ingestion. The AUC for the *Residual* and *Muscle Free* fractions was 1·3 and 1·9-fold higher in DLM fed fish than in fish fed MHA diet, respectively, indicating a higher bioavailability of the Met source in the fish fed the DLM diet (Table 5).

**Fig. 6.**
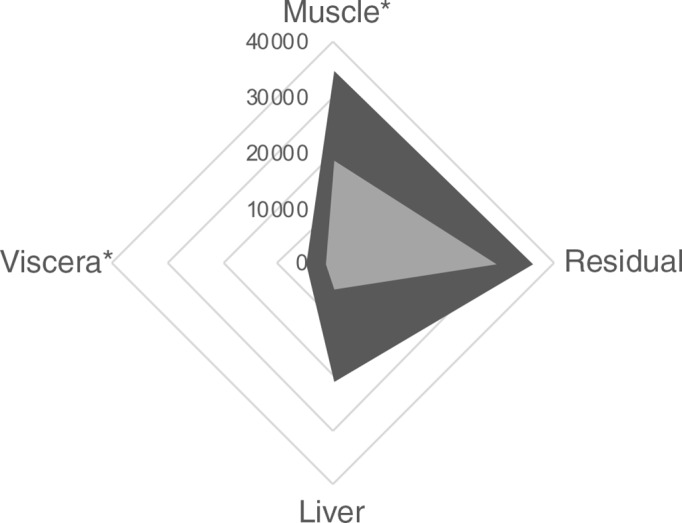
Radioactivity (DPM) in the *Viscera*, *Liver*, *Residual* and *Muscle Protein* fractions at 6 h after tube-feeding experimental diets labelled with ^14^C-dl-Met or ^14^C-MHA. Values are presented as means (*n* 6–7 fish for each diet). Asterisks denote significant differences (*P* < 0·05) between diets within the same compartment. 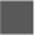, DLM; 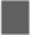, MHA.

At the end of the incubation period (6 h), a significantly higher amount of tracer was determined in the *Viscera* and *Muscle Protein* fractions from DLM fed fish (Fig. 6; *P* < 0·05). No significant differences between treatments were detected in *Liver* and *Residual Protein* fractions (*P* > 0·05). Regarding the *Others* fraction (lipids and other metabolites), there were no significant differences between treatments in all compartments (*P* > 0·05). The amount of tracer present in the *Liver Others* fraction was the lowest (333 and 222 DPM for fish fed the DLM and MHA diets, respectively) and the highest in the *Residual Others* fraction (2858 and 3037 for DLM and MHA fed fish, respectively).

## Discussion

The *in vivo* method using radiolabelled tracers (^14^C-dl-Met and ^14^C-MHA) was utilised to gain a deeper understanding on how different Met sources may affect the dietary Met utilisation by Nile tilapia juveniles. The amount of tracer found in the *Incubation Water* increased with time in both treatments; moreover, the analysis of the AUC from 1 to 6 h after ingestion revealed a higher amount of ^14^C-MHA than of ^14^C-dl-Met indicating a greater evacuation of unabsorbed MHA and/or higher catabolism of this Met source compared with dl-Met. The solubility of MHA-Ca in water is higher than that of dl-Met^(38)^; however, since the labelled pellets were placed directly into the fish oesophagus and were not in contact with water, the radioactivity present in the *Incubation Water* was the result of the evacuation of unabsorbed tracer. Similarly, in broiler chicks fed ^14^C-dl-Met or ^14^C-MHA a higher amount of tracer was present in the excrements of ^14^C-MHA fed birds than in excrements of birds fed the ^14^C-dl-Met^(39,40)^. dl-Met and MHA uptake is mediated by Na-dependent transporters across the brush border membrane^(9,10)^; however, MHA is also partly transported by diffusion^(41)^. In rainbow trout, it has been demonstrated that MHA presents a slower intestinal absorption rate than dl-Met^(10)^. This indicates that MHA stays longer in the intestine being more prone to bacterial degradation^(42)^ that would lead to a further decrease in the intestinal absorption of MHA and its evacuation. Combined, these two factors may contribute to the increased amount of unabsorbed ^14^C-MHA relative to ^14^C-dl-Met in the *Incubation Water*.

In our study, *Viscera* and *Liver Free* fractions of DLM and MHA fed fish presented a similar cumulative bioavailability of ^14^C-dl-Met and ^14^C-MHA for the 6 h period. However, a comparison between the two dietary treatments revealed different patterns of tracer flux in these compartments throughout the *in vivo* trial. Fish fed the DLM diet presented a peak in *Liver Free* fraction 1 h after feeding, while no peak was observed during the 6 h time course for MHA fed fish. The d-isomer of dl-Met and l- and d-isomers of MHA need to be converted to l-Met to become available to the animal via a two-step process^(8,43)^; however, the l-isomer present in dl-Met is readily available. Therefore, the results from the *in vivo* metabolic trial suggest a faster availability of dl-Met to metabolic processes than of MHA.

In fish fed the DLM diet, the tracer was available to the *Residual Free* fraction (*Free* fractions in head, heart, kidney, bones and fins) faster than in MHA fed fish. Moreover, for the 6 h trial, the cumulative Met source bioavailability in *Residual* compartment was higher in DLM than in MHA fed fish. Although the liver is the major site of conversion of d-Met and of d- and l-MHA to l-Met, this process also occurs in the kidney^(8,39,44)^. This may explain the high amount of tracer found in the *Residual Free* fraction, as this compartment includes the kidney among other tissues. Other studies have reported higher amounts of ^14^C-dl-Met than of ^14^C-MHA recovered in the kidney of broilers^(39,40)^. In a study where chicken kidney homogenates were incubated with ^14^C-labelled l-Met, dl-Met or MHA, dl-Met was found to be the substrate most readily converted into the intermediate KMB, revealing that the majority of the KMB produced in the kidney would result from the oxidation of the d- and not the l-isomer of dl-Met^(44)^. Further studies are necessary to confirm if in fish the kidney plays an important role in the conversion of d- to l-Met.

After being converted, the Met originating from the different dietary Met sources is transported to the rest of the body to be utilised by the fish. As a consequence of the faster absorption and hepatic metabolism, 2 h after feeding a higher amount of tracer was observed in the *Muscle Free* fraction of DLM fish, implying that dietary Met is available earlier for utilisation when fish are fed this source. Also, cumulative Met bioavailability in *Muscle Free* fraction was higher in DLM than in MHA fed fish. Therefore, one can consider that Met is readily available for protein synthesis faster and for a longer period in DLM fed fish than in fish fed the MHA diet. In fact, 6 h after feeding, the amount of tracer found in the *Protein* fractions of all compartments of DLM fed fish was higher than in MHA fed fish, with significant differences detected in *Viscera* and *Muscle* compartments. This indicates that DLM fish exhibited a faster and more effective incorporation of Met into muscle protein. Although the amount of tracer present in the *Liver Protein* fraction of DLM fish was higher than in fish fed the MHA diet, the difference was not significant due to a high variability within treatments. The vital physiological functions of the liver in detoxification, protein synthesis and digestion, constantly producing metabolites, may account for this variability.

In both dietary treatments, the amount of tracer retained in the lipid fraction, the major component of the *Others* fraction, was very low. Metabolic studies in gilthead seabream^(26,45)^ and Senegalese sole^(46)^ using a ^14^C-amino acid mixture have previously demonstrated that absorbed amino acids were preferentially used for protein synthesis and only a small proportion were converted into lipids.

At the end of the growth trial, the content of hepatic free amino acids and one-carbon metabolites was determined in fish fed control and supplemented diets. The content of total hepatic free amino acids in fish fed the DLM diet was significantly higher than in fish fed the REF or the MHA diets, due to an increase in both indispensable and dispensable amino acids. Since these are not postprandial results, it indicates a higher bioavailability of amino acids for metabolic purposes in Nile tilapia fed diets supplemented with DLM. Met supplementation caused an increment of free Met in liver, independently of the source. Met dietary supplementation studies in Nile tilapia^(47)^ and Atlantic salmon (*Salmo salar*)^(48,49)^ were unable to observe a similar effect of dietary supplementation in hepatic Met levels, possibly due to the fact that these studies report postprandial results unlike the present study where fish were sampled 24 h after feeding, hence the result of basal metabolism. The increase in free Met levels in liver indicates a higher availability for protein synthesis, transmethylation reactions and antioxidant defence in fish fed supplemented diets (DLM and MHA diets) compared with fish fed the non-supplemented diet (REF diet). In the present study, DLM and MHA fed fish presented similar levels of hepatic Met, SAM and SAH, while homocysteine was significantly lower in fish fed the DLM diet in comparison with the other dietary treatments. This suggests that the different Met sources follow different metabolic pathways. In neonatal pigs, to meet Met requirements, protein synthesis is favoured over Met transmethylation and the Met pool is conserved by increasing homocysteine production and suppressing transsulphuration^(50)^. In the present study, the hepatic free amino acid analysis indicates that fish fed MHA and REF diets probably remethylate homocysteine back to Met, with the addition of a methyl group from trimethylglycine. On the contrary, DLM fed fish seem to divert Met to the transsulphuration pathway, resulting in a significantly higher hepatic taurine content in DLM compared with MHA fed fish. Similarly, feeding Atlantic salmon with soy-based diets supplemented with dl-Met also resulted in an increase in the transsulphuration pathway and consequently higher hepatic taurine content^(48)^. These results indicate a stimulation of the transsulphuration pathway in fish fed the DLM diet.

DLM and MHA fed fish had a similar intake of dietary Met, cysteine and taurine. The experimental diets were soy-based, hence low in taurine. In fact, taurine content in all diets was below 0·1 g/kg. Taurine is an end product of Met metabolism and of the transsulphuration pathway. Therefore, although Met and cysteine hepatic levels were similar in fish fed DLM and MHA diets, a higher hepatic taurine content was found in the former, probably due to a higher availability of Met, as indicated by the metabolic trials. Higher hepatic taurine content is beneficial for the fish as taurine is involved in numerous physiological functions. In fish, taurine plays important roles in bile salt formation^(51,52)^, lipid digestion^(27)^, osmoregulation^(53)^ and antioxidant defence^(54–56)^ and it also increases amino acid retention^(27,57)^. In Nile tilapia, it has been demonstrated that dietary taurine supplementation improves growth performance^(58)^. In the present work, taurine status in DLM fed fish might have partially contributed to the improvement in growth performance observed in the growth trial. Diet supplementation with dl-Met increased fish body weight when compared with REF and MHA fed fish. Relative to the REF diet, DLM diet produced more 214 g in biomass gain, whereas MHA diet produced only more 124 g (on tank basis). This indicates that on equimolar basis MHA is only 58 % as efficient as dl-Met in terms of biomass gain. dl-Met supplementation improved feed conversion and protein efficiency ratios when compared with the basal diet (REF), while MHA fed fish presented intermediate results. These results are in agreement with previous studies in fish^(13,16,17,59)^, demonstrating that dl-Met supplementation improves growth and promotes protein accretion more efficiently in Nile tilapia.

In the metabolic trial, it was established that the distinct Met sources are utilised differently by Nile tilapia juveniles in the short term. *Residual* and *Muscle Free* fractions shown higher availability of Met in DLM than in MHA fed fish and 6 h after feeding a greater amount of tracer in the *Muscle Protein* fraction was found in the former. These differences were also reflected in the long term. At the end of the experimental period, protein retention in DLM fed fish was higher than in fish fed the MHA diet. Protein and total Met whole-body content were also higher in fish fed the DLM diet than in fish from the MHA group. The differences found between DLM and MHA fed fish in total Met content and availability explain the differences in protein content, reinforcing that Nile tilapia ultimately utilises dl-Met more efficiently for protein deposition than MHA.

The augmented protein retention was ultimately reflected in the N balance. All diets were isonitrogenous and feed intake was similar in all treatments, resulting in similar N intake among treatments. However, DLM fed fish presented the highest N gain and the lowest N losses, indicating that these fish were more efficient in retaining N than MHA fed fish. Similar results have been reported in studies performed with rainbow trout juveniles^(16)^, where the relative bioavailability of MHA was compared with dl-Met by dose–response trials regarding growth performance and nutrient retention. Lower N losses are related to higher protein digestibility and/or lower catabolism, resulting in lower N release to the environment. The *in vivo* metabolic trial revealed that the amount of tracer present in the *Incubation Water* increased with time and was lower in fish fed the DLM diet than in MHA fed fish. Consequently, in the long-term trial this is reflected in the N balance, indicating that dietary dl-Met supplementation contributes to a reduction in the environmental impact of Nile tilapia farming.

In conclusion, dietary Met sources influence Met absorption and utilisation in Nile tilapia juveniles. The *in vivo* study indicated that dl-Met is more retained than MHA probably due to a faster absorption rate as well as a greater availability of free Met in the tissues to be utilised by Nile tilapia. Additionally, Met from the different sources appears to follow distinct metabolic pathways; while Met from the DLM seems to be transsulphurated, Met from MHA and REF diets is probably remethylated to Met to maintain the free Met pool. In the long term, dietary dl-Met supplementation of soy-based diets improved growth performance and N retention in Nile tilapia, reducing the environmental impact and contributing towards a more sustainable industry.
